# A Multicenter Registry Experience for Suicidal Behaviors in Iran (2019-2022)

**DOI:** 10.34172/aim.28558

**Published:** 2024-07-01

**Authors:** Marjan Fathi, Seyed Kazem Malakouti, Mohsen Rezaeian, Kourosh Sayehmiri, Abbas Sheikhtaheri, Ida Ghaemmaghamfarahani, Ahmad Hajebi, Behrooz Ghanbari, Farnaz Etesam

**Affiliations:** ^1^Mental Health Research Center, Iran University of Medical Sciences, Tehran, Iran; ^2^Geriatric Mental Health Research Center, School of Behavioral Sciences and Mental Health, Iran University of Medial Sciences, Tehran, Iran; ^3^Department of Epidemiology and Biostatistics, Occupational Environment Research Center, Rafsanjan Medical School, Rafsanjan University of Medical Sciences, Rafsanjan, Iran; ^4^Psychosocial Injuries Research Center, Faculty of Health, Ilam University of Medical Sciences, Ilam, Iran; ^5^Department of Health Information Management, School of Health Management and Information Sciences, Iran University of Medical Sciences, Tehran, Iran; ^6^Department of Psychology and Educational Sciences, Islamic Azad University, South Tehran Branch, Tehran, Iran; ^7^Research Center for Addiction & Risky Behaviors (ReCARB), Department of Psychiatry, Iran University of Medical Sciences, Tehran, Iran; ^8^Trauma and Injury Research Center, Iran University of Medical Sciences, Tehran, Iran; ^9^Psychosomatic Research Center, Department of Psychiatry, Imam Khomeini Hospital Complex, Tehran University of Medical Sciences, Tehran, Iran; ^10^Baharloo Hospital, Tehran University of Medical Sciences, Tehran, Iran

**Keywords:** Registry program, Self-harm, Suicide, Suicide attempt, Suicidal behavior

## Abstract

**Background:**

Considering the limited information on suicide determinants, especially in low- and middle-income countries, the establishment and promotion of a suicide registration system are among the prominent strategies for suicide prevention programs around the world. The multicenter suicide registry is designed to collect standardized data from the two provinces of Iran according to the latest World Health Organization (WHO) guidelines.

**Methods:**

The Suicidal Behavior Registration Program is a multicenter study designed in five stages, including literature review, infrastructure establishment, database design, training, data analysis, and examining opportunities and challenges. The research samples cases of suicide attempts and self-harm from hospitals in the provinces of Tehran and Ilam.

**Results:**

The multicenter suicide registration program was carried out for 8 months in the two provinces of Tehran and Ilam. During the study period, data of 1382 people were registered, of which 7 cases in Ilam resulted in death. The study uncovered significant differences in socio-demographic, psychological status, and suicide characteristics in the two provinces.

**Conclusion:**

The design and implementation of the suicide registration program help researchers and policymakers make more innovative and effective interventions to prevent suicide by creating a comprehensive database of suicidal behavior determinants.

## Introduction

 Suicide is one of the significant public health concerns worldwide and affects all age groups.^1^ According to the World Health Organization (WHO), more than 700 000 people lost their lives due to suicide in the world in 2019.^2^ Furthermore, the number of suicide attempts is 20 times higher than the estimated number of suicides leading to death, globally.^3^

 Iran has reported the highest rate of suicide in the Eastern Mediterranean region (EMR) in recent decades.^4^ In 2020, death rate due to suicide was documented at about 6.6 per 100 000 attempts in Iran. The rate of suicide has increased by about 1.5 per 100 000 people in the last five years.^5^ The suicide attempt rate has also increased from 100.72 per 100 000 to 121.33 per 100 000 in Iran from 2015 to 2020.^5^ According to similar statistics, suicide and related deaths are growing in Iran.^4,6^ This rate is higher in some western provinces, including Ilam.^5,7^ In addition, studies have shown that a history of one or more suicide attempts and previous self-harm is one of the most important predictors of suicide attempts and suicide-related deaths.^8,9^ A review of 48 studies by Forte et al estimated the cumulative distribution of suicidal behavior at 26.4% within the initial month and 73.2% within 12 months of discharge.^10^ Geulayov et al found that patients who are referred to the hospital for self-harm are at probable risk of committing suicide again, particularly after hospital admission.^11^

 Given that suicide, suicide attempts, and self-harm have broad dimensions, immediate actions to implement suicide prevention interventions and reduce mortality are among the indicators of the United Nations Sustainable Development Goals (SDGs). Thus, the implementation of the WHO Mental Health Action Plan (2013‒2030) is a priority.^12^ Therefore, surveillance of suicidal behaviors will lead to effective suicide prevention programs. Besides, monitoring suicidal behaviors in the target population can be achieved through access to practical and factual data. However, there is evidence indicating that many countries have severe challenges accessing this data.^12^ Therefore, registering and improving the quality of suicide and suicide attempt data are necessary for governments to design effective interventions and strategies to prevent suicide, as stated in the WHO report.^13^

 Most suicide data registry programs provide limited and partial information that may offer fewer requirements for preventive measures and effective interventions.^13^ Suicide registry programs in hospitals in a particular country develop some knowledge of suicide identification and provide valuable information for promoting evidence-based interventions and suicide prevention programs.^13^ Accordingly, the WHO published a comprehensive guideline for the establishment and promotion of suicide, suicide attempts, and self-harm registration programs in 2014 and 2016.^3,13^ To this end, a few countries have launched suicide registration programs. For example, Ireland, Australia, Taiwan, and the United Kingdom have implemented suicide hospital-based registries at national and regional levels.^14-16^ However, most low- and middle-income countries have not yet planned to register suicidal behavior in hospitals.^13^

 The suicide registry system was designed and launched by the Ministry of Health in Iran in 2008.^17^ Since then, the data have been collected from the hospitals and sent to the health deputies of medical sciences universities to be uploaded to the main database. These data include the demographic characteristics of suicide attempters and the type of attempts (method, place of suicide, time, and outcome).^5,17^ However, due to limited resources and time constraints, access to clinical and diagnostic variables, family, and other components mentioned in the World Health Organization guidelines is not possible in the national suicide registration program.

 The suicide registry program in Tehran and Ilam provinces has been designed based on WHO’s “Preventing Suicide, A Manual for Case Registration of Suicide and attempted suicide “ versions 2014 and 2016.^13,14^ One of the features of the project is to assess more details of suicide characteristics in the two different provinces with different cultures.

## Aims

 The registry program aims to enhance understanding of suicidal behaviors, identify related factors, pinpoint high-risk groups, and aid in developing suicide prevention programs. It does so by improving data quality, identifying risk factors in vulnerable populations, and monitoring trends and emerging risk factors to enhance prevention efforts.

## Materials and Methods

###  Study Design

**Figure 1:**
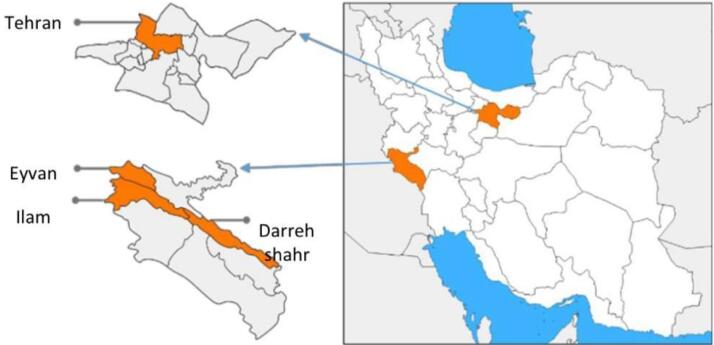
Geographical Locations of Data Collection in Iran

 The city of Tehran, in the Tehran province with 8 737 510 population, is the capital of Iran. It has 66% of the total population of the Tehran province.^18^ The suicide rate in the Tehran province has increased by 3.3 per 100 000 people.^5^ Ilam is one of the provinces in western Iran. The cities of Ilam (the capital of the province), with 235 144 people, Eyvan (49 491 people), and Dareh Shahr (43 708 people), include 55% of the total population of the Ilam province.^18^ Suicide deaths in Ilam have increased from 16.3 per 100 000 in 2015 to 16.56 per 100 000 in 2020.^5^ Figure 1 shows the geographical situation of Tehran and Ilam provinces, which are the places where registry programs were implemented in Iran.

###  Study Participants

 Potential study participants in this study were suicide attempters admitted to the emergency departments of the four main referral hospitals in the two provinces. The rate of referral of suicide cases, the interest and cooperation of hospital officials, and the availability of necessary hardware and software facilities were the selection criteria.

 Inclusion criteria for participants:

- Individuals admitted to an emergency department in the selected hospitals and assessed by physicians as likely to have intentional self-injury according to the 10th revision of the International Classification of Diseases (ICD-10), entitled “Intentional self-harm” (codes X60-X84).^19^
- Those who gave written consent to participate in the study.

Exclusion criteria for participants:

- Death before admission to the hospital.
- Accidental and excessive consumption of alcohol or drugs without suicidal intent.
- Accidental and overuse of prescription or over-the-counter medications and any other unintentional harms.
- Self-harm behaviors without the purpose of death (self-mutilation).
- Existence of severe mental disorder in such a way that the person is not aware of the deadly consequences of his action.
- Lack of informed consent for participation in the program.

###  Recruitment and Task Schedule

**Figure 2:**
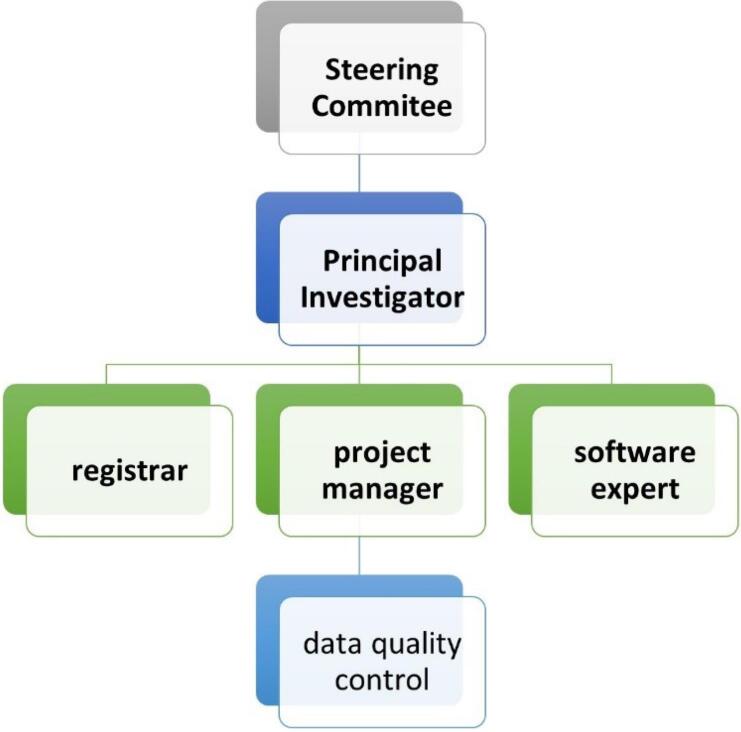
Overview of the Management Structure for Collecting Data

 The multicenter suicide registry program is implemented in a concerted effort by Iran, Tehran, and Ilam Universities of Medical Sciences in Iran. The steering committee consisted of 9 experts in psychiatry, psychology, health information management, epidemiology, biostatistics, and psychiatric nursing in Iranian universities of medical sciences that lead the program. The steering committee has identified the stakeholders, defined objectives, provided the project fund, prepared standard operating procedures, monitored and evaluated the performance, and controlled data quality. The steering committee also supervised software design, data entry, and data analysis (performed by an executive team of information technology (IT) experts). A member of the steering committee, as the project manager, was responsible for interacting with universities, providing hardware and software infrastructure, recruiting and training registrars, and monitoring the registration to ensure that the implementation followed the instructions. The project manager also reviewed the data registration process and quality control and reported to the steering committee regularly. Figure 2 shows the management structure of the registration program.

###  Tools 

 For collecting and recording suicide, suicide attempts, and self-harm data, a data registration form, data dictionary, necessary questionnaires (introduced below), and data registration guidelines were developed. The steps followed to prepare the required data are:

(a) A list of data was compiled using different sources: Preventing suicide: a manual for case registration of suicide and attempted suicide, WHO, 2014,^3^ Practice manual for establishing and maintaining surveillance systems for suicide attempts and self-harm, WHO, 2106^13^; Supre-miss, the WHO Multisite Intervention Study on Suicidal behaviors, 2009^20^; International Standard Classification of Occupations (ILO)^21^ adapted to Iran Job Classification; Place of occurrence of the external cause Y92 (ICD10)^22^; self-harm Code Table (ICD10)^19^; Risk factors associated with suicidal ideation and suicide attempts in Bhutan^23^; Health Survey Northern Ireland Questionnaire, 2018/2019^24^; England Registry Data, 2019; Scotland Registry Data, 2019^25^; Japan Registry, 2017^26^; Iran Ministry of Health Suicide Questionnaire, 2019^5^; Ilam s = Suicide Registry Questionnaires, 2018^27^, Charlson Comorbidity Index^28^.
(b) The minimum data required was finalized by the steering committee.
(c) The data registration form was completed on a trial basis at Baharloo Hospital in Tehran for a month and then completed after the amendments.

Due to the relationship between impulsivity, anger intensity, and suicidal behaviors,^29,30,31^ the patients were examined with three valid questionnaires. Finally, the registrars recorded the obtained scores.

 Tools for assessing the psychological status of the patient included:

- *Beck Scale for Suicide Ideation (BSS): *Suicide ideation is a significant risk factor for future suicide.^32^ This 19-item scale measures the severity and thoughts of suicide and a person’s intention and planning to attempt suicide.^33^ This scale was validated in Iran in 2004 and 2015. The reliability of the test is between 0.87 to 0.97 using Cronbach’s alpha and 0.54 with test-retest.^33,34^
- *Barratt Impulsiveness Scale (BIS-11): *Impulsivity is another significant risk factor for suicidal behaviors. The 30-item Barratt Scale is a tool to measure impulsivity.^35^ This scale was validated in Iran in 2012 by Javid et al. Cronbach’s alpha and its retest correlation coefficient were 0.81 and 0.77, respectively.^35^
- Spielberger’s* State-Trait Anger Expression Inventory-2 (STAXI-2): *This 57-item questionnaire includes six scales and five subscales and is a common and effective tool for measuring anger.^36^ In the study by Khodayarifard et al, the content validity of the test was reviewed and confirmed by experts. The items on the questionnaire were divided into three sections and measured the intensity of experience, the onset, and control of anger. Using Cronbach’s alpha, reliability coefficients in test components were between 0.6 and 0.93 in Iran.^37,38^

###  Registration Software

**Figure 3:**
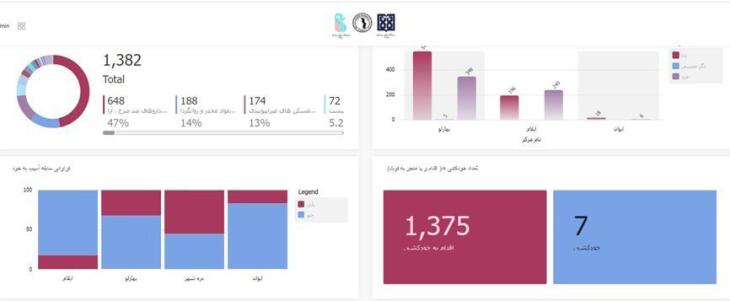
An Example of a Report Received From the Server Related to the Characteristics of Suicide Attempters

**Figure 4:**
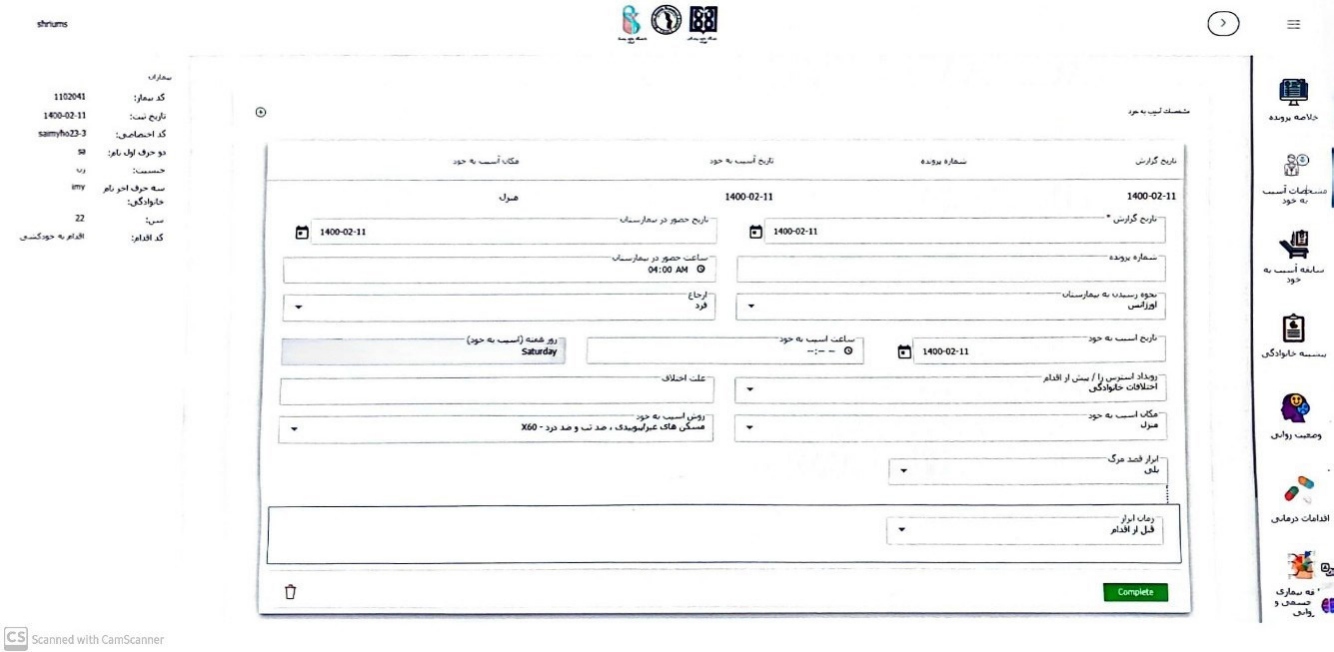
A Summary of the Recorded Cases of Attempted Suicide

 A private IT company developed the suicide registry data software, hosted by the Iran University of Medical Sciences. The web registration system allows real-time and secure data access, with the ability to add new variables during implementation and preventing duplicate cases. The software, developed using C# JavaScript in the VISUAL STUDIO programming environment, includes nine forms for data entry and access based on user roles. The software is accessible at https://shr.iums.ac.ir. Screenshots of the software are shown in Figures 3 and 4.

Figure 3 shows the method of self-harm based on ICD10 criteria in the areas covered by the suicide registry in the top left, the frequency of registered suicide attempts by gender and area in the top right, a comparison of suicide attempts in covered areas in the bottom left, and the number of registered suicide cases by the result of the action in the bottom right.

 In Figure 4, the right dashboard shows case details such as self-injury, family history, mental status, and medical procedures. The left side displays patient codes, registration dates, and demographic information. The central part shows self-harm profiles, family history, history of self-harm, and mental state of the registered suicide cases.

###  Establishment 

 After preparing the infrastructure and guidelines, the project manager selected psychologists with a master’s degree based on their resumes and interviews. The selection criteria included IT skills, empathy in patient communication, and collaboration with hospital staff. The registrars were then trained on program goals, registration software, data forms, and data collection methods in a three-hour workshop involving lectures and role-playing.

 The registration program was piloted and any potential issues were addressed and fixed. The project manager monitored data entry online and registrars completed weekly monitoring forms for the first three months to ensure accuracy and quality. A form was designed for daily registration in the emergency room and department to improve data collection and allow for performance monitoring. Other strategies, such as field visits, online meetings, and a virtual group, were used to facilitate communication between the project manager and registrars for better program monitoring.

###  Data Collection 

 Suicide attempt survivors admitted to the emergency department received critical care from emergency physicians. After confirming the attempters’ consciousness, registrars requested their consent to participate in the study. Both the survivors and their families provided demographic information and details on suicide history. Hospital psychiatrists and psychologists evaluated the patients’ mental status. Data on their physical health, medical treatments, and instances of self-harm were gathered from medical records and promptly recorded online. In case of any issues with real-time data entry, like internet disruptions, registrars had a one-week window to input the data. Follow-up calls were arranged weekly for the first-month post-discharge, transitioning to monthly calls for a year thereafter.

###  Data Quality Control

 Each patient was given a unique code to prevent duplicate registrations. Mandatory fields were specified in the software to ensure complete information. Registrars were required to use instructions, a data dictionary, and validation rules for consistency. Data was reviewed daily by the registration supervisor, and registrars received feedback on any issues. Discrepancies had to be resolved within 24 hours. The executive manager reviewed the data monthly and reported to the steering committee every three months.

###  Statistical Analysis

 The collected data were studied descriptively and inferentially by the SPSS statistical software version 26. The data were analyzed using descriptive statistics (measures of central tendency, variability spread, and frequency distribution). Next, the chi-square coefficient method was used to compare the differences between the two provinces, and the significance of the differences was reported as a *P* value. Finally, the logistic regression method was used to determine predictor variables. The results were reported quarterly, six-monthly, and annually to the Research Vice-Chancellor of the executive universities.

## Results

 In the initial phase of the study, data from 1382 individuals, with 65.6% residing in Tehran and 34.4% in Ilam, were collected and monitored online for over eight months. Of these, 771 cases (56%) were women, 606 cases (43.5%) were men, and 5 cases (0.3%) were transgenders. Of this number, seven cases (about 0.5%) died after reaching the hospital, including two women (0.1%) and five men (0.3%). All the deceased cases were recorded in the Ilam province.

 In the Tehran province, the data collected was 1.9 times larger than Ilam province. The ratio changed in equal from March to the end of project.

 The suicide rate in the Tehran province shows a higher percentage in women (60.7%) compared to men (38.8%), with a 1.5-fold difference. In the Ilam province, the rate of suicide attempts among men was recorded at 53.4%, which is 1.2 times higher than that of women (46.6%).

 Moreover, 77% of data registration in the Tehran province occurred in the morning, while 23% took place in the afternoon. In the Ilam province, the morning-to-afternoon patient registration ratio was 96 to 4. The number of individuals who did not respond to the questionnaire in Ilam exceeded that of Tehran by 16.5 times. Notably, a history of suicide among family and friends emerged as a significant indicator for previous suicide attempts and psychiatric hospitalizations. Moreover, the use of non-narcotic analgesics and the presence of physical ailments were identified as factors predicting psychiatric hospitalizations. Seeking treatment at a psychiatric facility, receiving pharmacological and psychiatric interventions, and having a history of suicide were also recognized as predictors for suicide attempts.

## Discussion

 Much more than any other field of medicine, research on suicide and its outcomes are shaped by global or regional alerts.^39^ Reliable information on suicide rates is complicated to obtain. However, the rate of suicide attempts is almost impossible to ascertain, especially because suicide attempts by the health care system.^40,41^

 The multicenter suicide registry project was developed to create a comprehensive and adaptable system for studying various factors influencing suicide. It allows for multi-center studies at national and international levels and provides a platform for conducting suicide research. The system was designed to receive specific regional suicide data and enabled real-time data collection to improve performance and operational efficiency. The evaluation of the registry program emphasized the significance of consistent, top-notch data as a crucial aspect of a system aimed at tracking trends and furnishing data for policy-making, planning, and prevention efforts. It also allowed for identifying trends in real-time and making dynamic changes as needed.

 The analysis of this data uncovered significant disparities in socio-demographic characteristics, psychological and psychiatric diagnoses, and suicide trends between the two provinces. The difference between the findings of the two provinces shows how much suicide depends on socio-cultural situations. To refer to the findings, cultural restrictions and social stigma of suicide should be taken into consideration, especially in the Ilam province. Not admitting to attempting suicide can underreport suicide numbers in both provinces, especially in the Ilam province and especially among women. The difference in the way to get to the hospital, and the difference in the use of medical services, can be a testimony to the effect of the social stigma of suicide and the lack of disclosure of the suicide attempt by the individual or the family, which is effective on the registration results.

 Comparing the quarterly and final research reports revealed limitations in generalizing the results to other hospitals and regions within the two provinces. Improved accuracy in data collection is crucial for obtaining reliable results. While the current findings offer insights into registered cases, they cannot be broadly applied to the entire two provinces, emphasizing the need for regional suicide prevention efforts. Recording suicide and attempted suicide cases is essential for enhancing prevention, diagnosis, and intervention strategies.^39^ The WHO consistently publishes the most reliable data on suicide rates, with information from 194 countries suggesting that suicide rates are influenced by economic, social, cultural, and environmental factors, as well as by age and gender.^40^ Globally, suicide rates have risen among individuals with chronic physical and mental health conditions, substance abuse issues, and those with a history of suicide attempts. Data quality worldwide is often moderate to low due to underreporting and detection challenges, resulting in limited information on suicide attempts, which are likely significantly more frequent than suicides.

 Several constraints of this assessment need acknowledgment. Stakeholders noted that the quality of data inputs and outputs significantly influenced assessing the system’s efficiency, acceptance, and longevity. However, concerns were raised about data quality, especially regarding potential variations in information from external sources. While stakeholder interviews may have biases, suicide prevention experts, with a vested interest in surveillance systems, were intentionally chosen.

 Data quality challenges intersect with observations on usefulness and timeliness. The assessment highlighted that information promptness could hinder data entry, particularly in complex cases, and the speed of information dissemination, especially for comprehensive records. A key research limitation was staff availability only during office hours at the hospital, resulting in data loss from evening, night, and holiday shifts. Ilam had a significantly higher non-response rate to questionnaires compared to Tehran. Factors such as suicide stigma, cultural issues, and using hospital staff as registration experts in Ilam’s first quarter contributed to incomplete questionnaires due to lack of motivation or opportunity.

 In this registration program, we have only been able to register suicide attempts and deaths due to suicide in specific hospitals. As a result, cases from general hospitals and private centers remain unregistered. An additional concern involves the Forensic Medicine Organization, where administrative obstacles hinder the reporting of suicide statistics to other organizations. The collaboration between these entities is limited, lacks structure, and is unaided. This leads to lack of access to many suicide cases within the healthcare system.

 One of the most important issues that we have faced in continuing registration is the problem of registration fees and receiving funds to provide human resources and pay expenses. Based on a prospective study in Canada in 2010, the total annual cost of implementing suicide prevention programs reached CAD 23, 982 293 in Quebec, Canada. These costs included suicide prevention program costs, direct medical and non-medical costs, police investigation and funeral costs, and indirect costs associated with loss of productivity. The highest cost pertains to follow-up and psychotherapy for suicide survivors from suicide attempts.^12^ According to the WHO economic analysis in 2019, prohibition of hazardous pesticides, school-based social-emotional learning programs, and adolescent suicide prevention were cost-effective interventions at the population level.^39^ Based on these findings and recommendations, accurate knowledge about the characteristics of suicide can play an essential role in promoting mental health programs and reducing the economic and psychological burden on society.

 The multicenter suicide registry program facilitated the comparison of suicide and self-harm trends between the two provinces and other regions. It also enabled the collection of demographic information and risk factors associated with suicide and self-harm in comparison to other areas. By obtaining precise data on suicide methods, this program assists policymakers in limiting access to suicide means, aligning with the World Health Organization’s suicide prevention strategies. Additionally, analyzing the methods and frequencies of pre-attempt suicide aids in evaluating the impact of previous suicide attempts and self-harm on re-attempts. Moreover, the multicenter suicide registry program contributes to suicide prevention efforts by exploring psychological and psychiatric factors during hospitalization. It enhances understanding of the influence of cultural, social, and economic factors on suicide and self-harm. We believe this registry program empowers mental health and suicide prevention experts to develop tailored prevention initiatives based on suicide trends in diverse populations. Therefore, implementing a suicide registration system in the Ilam and Tehran provinces, alongside establishing an electronic research platform on suicide, will effectively advance suicide prevention strategies, enhance mental well-being, and reduce long-term healthcare costs for society.

 Launching a suicide registration program requires collaboration, financial support, and teamwork. Challenges include changing budgets, suicide stigma, and confidentiality concerns. However, the program provides an opportunity to gain insight into suicide patterns and evaluate changes during the COVID-19 pandemic.

 The study emphasizes the need to consider socio-cultural nuances in developing effective suicide prevention strategies and stresses the importance of precise demographic and clinical information to guide interventions. The program’s strengths include its large sample size and adherence to WHO guidelines, which are expected to enhance knowledge of suicide and inform prevention policies.

 To enhance the registration program’s quality, several beneficial measures can be implemented:

- Addressing incomplete questionnaire responses and data loss in Ilam by boosting participant engagement and ensuring thorough data collection processes.
- Reducing the 30% data loss from single-shift data collection by spreading it across multiple shifts or using additional methods.
- Understanding and addressing the 16.5-fold higher non-response rate in Ilam compared to Tehran by considering socio-cultural factors.
- Managing the COVID-19 impact by supporting hospital staff, addressing infection fears, and maintaining motivation. Monitoring and addressing challenges in utilizing hospital resources for COVID-19 patients and its impact on hospital visits for self-harm cases are crucial for program effectiveness.
- Generalizing findings would require more resources. The suicide registry program should align with global best practices, emphasizing a comprehensive data collection approach in Tehran and Ilam to improve prevention strategies.
- Including detailed expenses for operations, data collection, staff training, and maintenance for the purpose of budget transparency. Regular budget reviews and adjustments based on evolving needs are vital for program success.

## Conclusion

 The design and implementation of the suicide registration program help researchers and policymakers make more innovative and effective interventions to prevent suicide by creating a comprehensive database of suicidal behavior determinants.
